# Supplementary material for: Wealth-based inequality in the exclusive use of hygienic materials during menstruation among young women in urban India
Source: PLoS One. 2022 Nov 29;17(11):e0277095. doi: 10.1371/journal.pone.0277095 (PMC9707774; doi:10.1371/journal.pone.0277095)
Supplement: S1 Table — (DOCX) [file pone.0277095.s001.docx]

**S1 Table. Erreygers normalised CI for Indian states and UTs in the exclusive use of hygienic materials among urban women aged 15-24 years in India, NFHS-5, 2019-21.**

| **States** | **No. of observations** | **Erreygers Normalized CI** | **Standard error** | **p-value** |
| --- | --- | --- | --- | --- |
| Andaman & Nicobar Islands | 145 | 0.068 | 0.045 | 0.132 |
| Andhra Pradesh | 842 | 0.148 | 0.036 | <0.001 |
| Arunachal Pradesh | 1343 | 0.084 | 0.026 | 0.001 |
| Assam | 1209 | 0.437 | 0.031 | <0.001 |
| Bihar | 1869 | 0.412 | 0.025 | <0.001 |
| Chandigarh | 221 | 0.203 | 0.048 | <0.001 |
| Chhattisgarh | 1757 | 0.303 | 0.027 | <0.001 |
| Dadra & Nagar Haveli and Daman & Diu | 443 | 0.027 | 0.046 | 0.564 |
| Goa | 327 | 0.255 | 0.060 | <0.001 |
| Gujarat | 3007 | 0.294 | 0.020 | <0.001 |
| Haryana | 1987 | 0.201 | 0.019 | <0.001 |
| Himachal Pradesh | 218 | 0.249 | 0.062 | <0.001 |
| Jammu & Kashmir | 1184 | 0.425 | 0.031 | <0.001 |
| Jharkhand | 1771 | 0.315 | 0.026 | <0.001 |
| Karnataka | 2596 | 0.311 | 0.020 | <0.001 |
| Kerala | 1191 | 0.198 | 0.032 | <0.001 |
| Ladakh | 149 | 0.281 | 0.092 | 0.003 |
| Lakshadweep | 250 | -0.098 | 0.059 | 0.097 |
| Madhya Pradesh | 3259 | 0.452 | 0.018 | <0.001 |
| Maharashtra | 3213 | 0.203 | 0.015 | <0.001 |
| Manipur | 604 | 0.322 | 0.045 | <0.001 |
| Meghalaya | 565 | 0.359 | 0.045 | <0.001 |
| Mizoram | 843 | 0.039 | 0.021 | 0.063 |
| Nagaland | 754 | 0.213 | 0.041 | <0.001 |
| NCT of Delhi | 3442 | 0.210 | 0.014 | <0.001 |
| Odisha | 1153 | 0.300 | 0.027 | <0.001 |
| Puducherry | 788 | 0.043 | 0.028 | 0.120 |
| Punjab | 1795 | 0.227 | 0.021 | <0.001 |
| Rajasthan | 3062 | 0.241 | 0.018 | <0.001 |
| Sikkim | 132 | 0.126 | 0.078 | 0.107 |
| Tamil Nadu | 2928 | 0.103 | 0.013 | <0.001 |
| Telangana | 1984 | 0.148 | 0.016 | <0.001 |
| Tripura | 344 | 0.376 | 0.059 | <0.001 |
| Uttar Pradesh | 6532 | 0.346 | 0.014 | <0.001 |
| Uttarakhand | 835 | 0.322 | 0.033 | <0.001 |
| West Bengal | 1819 | 0.366 | 0.023 | <0.001 |
| **India (total)** | **54561** | **0.302** | **0.004** | **<0.001** |
